# Loss of JCAD/KIAA1462 Protects the Lung from Acute and Chronic Consequences of Chronic Obstructive Pulmonary Disease

**DOI:** 10.3390/ijms25179492

**Published:** 2024-08-31

**Authors:** Ratoe Suraya, Tatsuya Nagano, Masako Yumura, Tetsuya Hara, Masaya Akashi, Masatsugu Yamamoto, Motoko Tachihara, Yoshihiro Nishimura, Kazuyuki Kobayashi

**Affiliations:** 1 Division of Respiratory Medicine, Department of Internal Medicine, Kobe University Graduate School of Medicine, Kobe 650-0017, Japan; dr.ratoesuraya@yahoo.com (R.S.); tnagano@med.kobe-u.ac.jp (T.N.); y_ym_mapie82@yahoo.co.jp (M.Y.); myamamot@med.kobe-u.ac.jp (M.Y.); nishiy@med.kobe-u.ac.jp (Y.N.); kkoba@med.kobe-u.ac.jp (K.K.); 2 Laboratory of Clinical Pharmaceutical Science, Kobe Pharmaceutical University, Kobe 658-8558, Japan; thara@kobepharma-u.ac.jp; 3 Department of Oral and Maxillofacial Surgery, Kobe University Graduate School of Medicine, Kobe 650-0017, Japan; akashim@med.kobe-u.ac.jp

**Keywords:** chronic obstructive pulmonary disease, JCAD, inflammation, angiogenesis, emphysema

## Abstract

Even with recent advances in pathobiology and treatment options, chronic obstructive pulmonary disease (COPD) remains a major contributor to morbidity and mortality. To develop new ways of combating this disease, breakthroughs in our understanding of its mechanisms are sorely needed. Investigating the involvement of underanalyzed lung cell types, such as endothelial cells (ECs), is one way to further our understanding of COPD. JCAD is a junctional protein in endothelial cells (ECs) arising from the KIAA1462 gene, and a mutation in this gene has been implicated in the risk of developing COPD. In our study, we induced inflammation and emphysema in mice via the global knockout of KIAA1462/JCAD (JCAD-KO) and confirmed it in HPMECs and A549 to examine how the loss of JCAD could affect COPD development. We found that KIAA1462/JCAD loss reduced acute lung inflammation after elastase treatment. Even after 3 weeks of elastase, JCAD-KO mice demonstrated a preserved lung parenchymal structure and vasculature. In vitro, after KIAA1462 expression is silenced, both endothelial and epithelial cells showed alterations in pro-inflammatory gene expression after TNF-α treatment. We concluded that JCAD loss could ameliorate COPD through its anti-inflammatory and anti-angiogenic effects, and that KIAA1462/JCAD could be a novel target for COPD therapy.

## 1. Introduction

Chronic obstructive pulmonary disease (COPD) is one of the most common diseases in the world and is accordingly rated as one of the most significant causes of mortality [1,2]. Even with the rapid progress of COPD research, current medications cannot sufficiently improve the prognosis of these patients [3]. Thus, the discovery of new knowledge to help understand the pathogenesis of COPD is important in finding novel therapeutic targets for this condition and improving its prognosis.

As the understanding of COPD pathophysiology has grown over the years, the traditional conception of COPD as simply a dysfunction of the lung epithelial cells has evolved into COPD as a multicellular failure that orchestrates the structural and functional decline seen in its clinical manifestations [4]. As such, an increasing number of cell types are being identified to possess a role in the progression of COPD. Some such cells are endothelial cells (ECs), a cell type previously thought to play a lesser role in COPD [5]. One particular structure in ECs that is currently being studied is the cell–cell junctional protein. Connecting junctional proteins are important not only as simple connectors between cells but also in maintaining functional integrity [6]. Over the years, many additional functions of junctional proteins have been identified, some of which relate to inflammation and angiogenesis [7]. Inflammation is a natural response of the body to various stressors and is influenced by a bevy of internal and external factors. In COPD, a continuous inflammatory reaction over a long period of time has been widely accepted as a central pathological process driving its development, and EC junction integrity is important in ensuring that the movement of immune cells into the site of damage will not be impeded or overpromoted [5,8]. Meanwhile, angiogenesis in the context of COPD is linked to the continuation of acute inflammation, a process that could be triggered by some of the immune cells recruited and/or cytokines produced during inflammation [9].

In 2017, a Genome-Wide Association Study (GWAS) identified polymorphism in the gene KIAA1462, encoding the protein JCAD (Junctional protein associated with Coronary Artery Disease) as a risk for COPD [10]. JCAD/KIAA1462 has previously been reported to be expressed in endothelial cells and acts in conjunction with VE-cadherin as an endothelial–endothelial junctional protein [11]. Additionally, this protein is known to be involved in angiogenesis and atherogenesis processes in pathological conditions [12,13]. However, no study has linked JCAD/KIAA1462 to the molecular process surrounding COPD, as suggested by the GWAS findings. 

In this study, we sought to analyze how JCAD exhibited protection against elastase-induced inflammation and further structural destruction. JCAD-deficient mice failed to develop a robust inflammatory response, which, in the long run, prevented any overt parenchymal loss and neovascularization. This was confirmed by in vitro studies, where in both human pulmonary microvascular endothelial cells (HPMECs) and lung epithelial A549 cells, JCAD knockdown prevented a wide range of inflammatory gene expressions, implicating JCAD in COPD development.

## 2. Results

### 2.1. Amelioration of Acute Inflammatory Response in JCAD-Deficient Mice after Elastase Instillation

**Figure 1:**
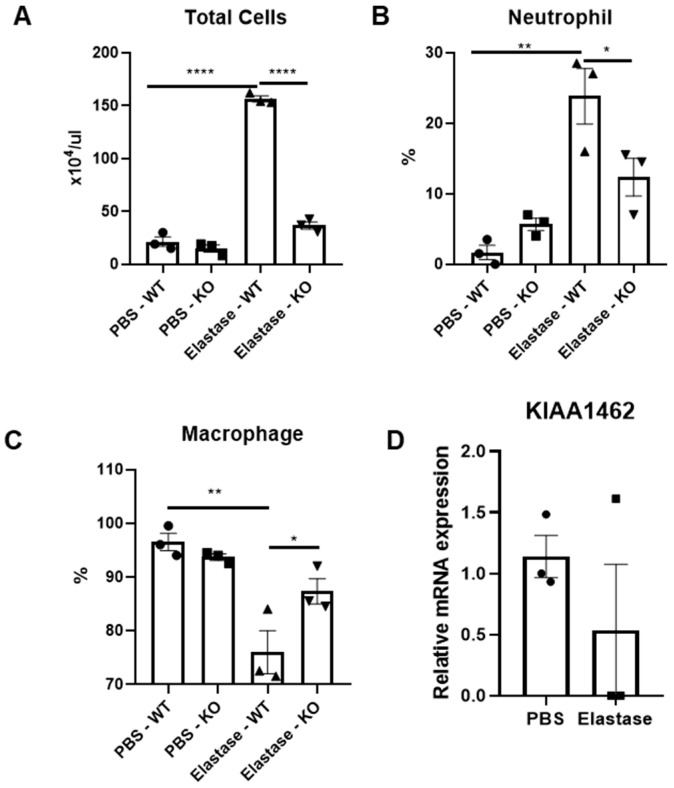
KIAA1462/JCAD knockout ameliorates acute inflammation after short-term elastase treatment. Total cell count (**A**), neutrophil percentage (**B**), and macrophage percentage (**C**) from BAL fluid of JCAD-KO or WT mice after 1 day of elastase or PBS treatment (*n* = 3–4 per group). (**D**) mRNA expression level of KIAA1462 in BAL cells of WT mice after 1 day of treatment of intratracheal elastase (*n* = 3 per group). Data are presented as mean ± S.E.M values. *, <0.05; **, <0.01; ****, <0.0005.

As ECs are important in inflammatory response initiation, we were first interested in what kind of effect JCAD-deficient ECs have on this particular process. To determine this effect, we utilized a one-day elastase induction of emphysema in mice to analyze the difference in the acute inflammatory process of the JCAD-KO mice compared to WT controls. Surprisingly, JCAD-less lungs exhibited dampened pro-inflammatory responses in the bronchoalveolar lavage (BAL) fluid compared to WT ones. This was shown by a reduced BAL fluid total cell count (Figure 1A) that was accompanied by reduced neutrophil recruitment to the lung without changes in the macrophages (Figure 1B). Interestingly, this was not accompanied by significant changes in the macrophage percentage in the BAL fluid (Figure 1C), while no changes in the KIAA1462 mRNA level were observed in the BAL cells (Figure 1D). As such, we concluded that there is an amelioration of an acute inflammatory reaction in JCAD-deficient lungs.

### 2.2. The Structural Integrity of the Lung Was Preserved in JCAD-KO Mice in an Emphysematous Model

**Figure 2:**
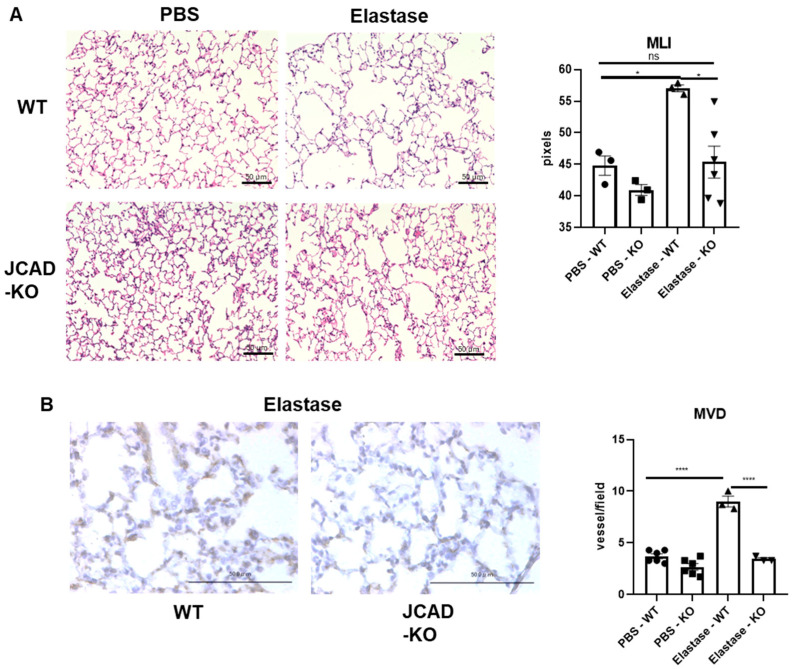
Lung structural integrity is preserved in mice lacking JCAD with normal vascularity. (**A**) Hematoxylin–eosin staining of lung sections from WT and JCAD-KO mice after 3 weeks of elastase in PBS treatment with mean linear intercept (MLI) quantitation (*n* = 3–6 mice/group). (**B**) CD-31 (cluster of differentiation-31) immunostaining of lung sections from WT and JCAD-KO mice after 3 weeks of elastase in PBS treatment with microvessel density (MVD) quantitation (*n* = 3–6 mice/group). Data are presented as mean ± S.E.M. *, <0.05; ****, <0.0005; ns, not significant.

Next, we further pursued the implications of a JCAD-deficiency-driven reduced inflammatory response of the lung after elastase administration under chronic conditions, which could be observed after 3 weeks of elastase administration in vivo. While in the WT lung, there is a marked destruction of the parenchyma causing emphysema, this elastase-induced destruction was ameliorated in the JCAD-KO lung (Figure 2A). Quantitative mean linear intercept (MLI) measurements confirmed this finding (Figure 2A). Again, we would like to determine the involvement of ECs in the chronic emphysema formation phase. JCAD is a known angiogenesis modifier, and considering the reduced inflammation in the early phase post elastase, we hypothesized that the inflammation-induced angiogenic process could also be affected by JCAD. Indeed, no adaptive angiogenesis, shown as an increased CD31-positive cell count through microvessel density (MVD) quantitation, was found in the JCAD-deficient lungs 3 weeks after elastase, while a robust increase in neovascularization could be easily found in the elastase-treated WT mice (Figure 2B).

### 2.3. Structural Integrity of the Lung Was Preserved in JCAD-KO Mice in the Emphysematous Model

**Figure 3:**
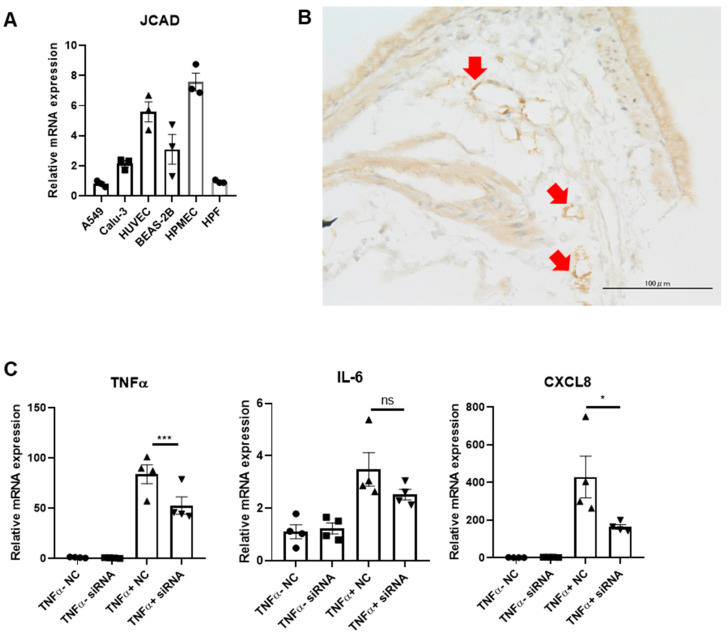
Pulmonary microvascular endothelial cells are important in the JCAD-deletion-derived reduction in pro-inflammatory and pro-angiogenic phenotypes. (**A**) mRNA expression levels of KIAA1462/JCAD in various lung cell lines (n = 3 per group). (**B**) JCAD immunostaining in lung section of WT mice; positive cells are indicated as red arrows. (**C**) mRNA expression levels of TNF-α, IL-6 (interleukin-6), and CXCL8 (CXC motif chemokine ligand 8) in HPMECs with JCAD knockdown before 6 h of TNF-α treatment (n = 4 per group). Data are presented as mean ± S.E.M. *, <0.05; ***, <0.005; ns, not significant.

We then utilized in vitro experiments to further elaborate how JCAD deficiency could prevent the worsening of COPD at the cellular level. After confirming the level of JCAD/KIAA1462 expression in various cell types present in the lung (Figure 3A) and in the lung sections of normal WT mice (Figure 3B), we picked out endothelial cells, which in our experiments were represented by HPMECs (human pulmonary microvascular endothelial cells). To mimic the pro-inflammatory environment seen in COPD pathology in an in vitro setting, we treated HPMECs with TNF-α (tumor necrosis factor-α) (or vehicle as controls) for 6 h after first silencing them with KIAA1462 siRNA or mock siRNA as controls. With the addition of gene-silencing treatments, TNF-α treatment interestingly failed to elicit a robust increase in pro-inflammatory cytokine mRNA expression in JCAD-less HPMECs compared to its control (Figure 3C). 

**Figure 4:**
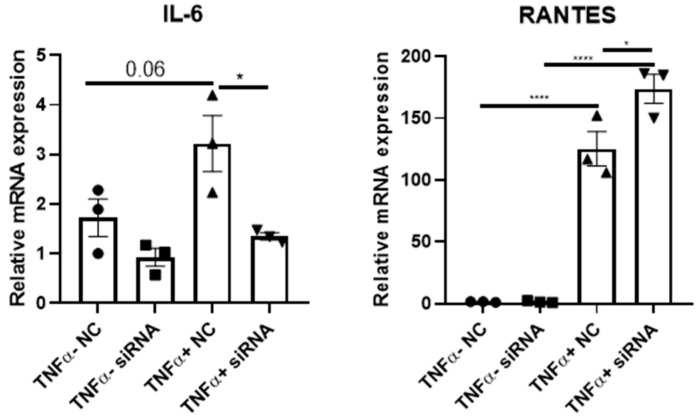
Epithelial-derived JCAD contributes to the inflammatory phenotype of the lung. mRNA expression level of IL-6 and RANTES in A549 cells after JCAD knockdown prior to 6 h of TNF-α treatment (n = 3 per group). Data are presented as mean ± S.E.M. *, <0.05; ****, <0.0005.

Lastly, while the level is low, KIAA1462 mRNA expression is still detectable in other lung cell types. Dysfunction in the epithelial cells is traditionally known as one of the main mechanisms of COPD, and the fact that JCAD is still detectable in this cell might imply that JCAD could also have a role beyond the ECs. As such, we analyzed whether the changes in pro-inflammatory cytokines are exclusive to the ECs or spread across the lung cell types. To do so, we performed similar TNF-α and siRNA treatments in A549 lung epithelial cells and found similar pro-inflammatory marker changes after JCAD/KIAA1462 silencing in these cells to HPMECs, most notably in IL-6, which is also an angiogenic factor (Figure 4). Taken together, we concluded that the loss of JCAD/KIAA1462 in the lungs could act beneficially in preserving its integrity through an anti-inflammatory and subsequent anti-angiogenic effect.

## 3. Discussion

Since its functional discovery as a protein that forms a complex with VE-cadherin in the endothelial cell–cell junction, JCAD/KIAA1462 has been found to exhibit angiogenesis-altering effects under different conditions, while the other functions of this junctional protein have not been extensively studied yet [11,12,13]. In our study, we showed for the first time how JCAD could potentially act in mediating the inflammatory and subsequent adaptive angiogenesis response in the lung during elastase-mediated emphysematous lung, mediated by a reduction in the pro-inflammatory phenotype of ECs of the lung.

Partly because its homozygous knockout mice are healthy and viable, the function of JCAD during physiological conditions has not been clearly elucidated yet [12]. However, when ECs are required to react to pathological stimuli, JCAD deficiency has been reported to be a modulating factor that could affect the response to said stimuli. For example, Hara et al. showed how introducing tumor cells in JCAD-KO mice could result in reduced tumor size and tumor neovascularization after tumor injection in vivo [12]. Another group showed how JCAD-less ECs could cause attenuated atherosclerotic plaque development [13]. It is clear that, at least after external insult, JCAD is involved in the cellular and tissue response.

An important response process that potentially involves ECs is inflammation [9]. Inflammation is widely known as a central mechanism in the development and progression of COPD [14]. While the role of EC dysfunction has been less defined in COPD pathobiology, evidence suggests that the pulmonary vasculature is an important link between the immune responses to chronic, repeated exposure to external pathogenic stimuli through its role [9,15]. Related to those, angiogenesis is another process that could be triggered both by the response of the stimuli or by the ensuing inflammatory actions of the cells, thus linking the lesser number of microvessels with the reduced inflammatory response and structural integrity shown in JCAD-KO mice [9,16].

In our study, we showed how JCAD deficiency could actually be beneficial in terms of preventing overt pathological inflammatory and angiogenic responses in COPD. Indeed, several studies have shown how EC junctional proteins could alter the neutrophil diapedesis process, the overall capability of the leukocyte migration process, and the correct organization of new vessels during angiogenesis [6,7,17]. These functions are possible not only via its ability to protect the structure and connections of ECs but also through the modulation of various molecular signaling pathways. We found that several important cytokines, such as TNF-α, IL-6, and the neutrophil-attracting chemokine CXCL8, could be significantly downregulated, which matches how there were significantly fewer neutrophils recruited in the BAL fluid of the mice [18,19]. While the specific mechanism linking JCAD to the alterations of those genes is beyond the scope of the current study, it is imperative that further studies are conducted on how the loss of part of a cell–cell junction protein could lead to the impairment of cytokine production in said cells. It would also be interesting to see whether this inflammation-, angiogenesis-, and subsequent lung-structure-altering effect of JCAD loss could also cause a different effect in other pulmonary conditions aside from COPD, such as pulmonary fibrosis, and further studies regarding this topic are warranted in the near future.

Furthermore, it is surprising to observe that non-EC cell types also express varying amounts of KIAA1462 mRNA levels, and even more astonishing is that the deletion of KIAA1462 could lead to an in vitro phenotype. This suggests that there is a possibility that JCAD could also function as a junctional protein beyond the known complex formation with VE-cadherin in the ECs. Notably, IL-6 seems to be a cytokine whose expression level is affected to varying degrees by the presence of JCAD regardless of cell type, warranting further investigations [20,21]. Further studies are required to elucidate the role of JCAD in non-ECs, but its inflammation and angiogenesis-mediating functions seem to be preserved in different cells.

Lastly, because JCAD knockout did not show any overt pathological phenotype in vivo, intervening JCAD as a therapeutic target might be a viable solution for future COPD therapy. While targeting the junctional proteins of ECs has not yet been fully explored in various biomedical fields, our results, coupled with other studies, might be able to serve as motivation to pursue the field of cell–cell connections as a novel way to treat COPD and many other conditions. 

## 4. Materials and Methods

### 4.1. Animal Study

JCAD-KO mice were generated as described in our previous manuscript [12], maintained in-house, and provided with food and water ad libitum and a 12 h dark–light cycle [12]. All experiments used 8-12-week-old mice. Littermate wild-type (WT) mice were used as controls for the experiments. The Ethics Review Committee approved all experimental animal protocols for Animal Experiments of Kobe University. As previously described, we applied intratracheal elastase (WAKO Fujifilm, Osaka, Japan) instillation (3 U/mice) or a PBS-instilled negative control to JCAD-KO or WT mice under intraperitoneal anesthesia (dexmedetomidine (40 μL; Maruishi Pharmaceutical, Osaka, Japan), midazolam (11 μL; Astellas, Tokyo, Japan), and butorphanol tartrate (13 μL; Meiji Seika Pharma, Tokyo, Japan)) [22]. For acute inflammatory phase analysis, mice were sacrificed 24 h post elastase instillation, while in the chronic emphysematous phase, mice were sacrificed 3 weeks post elastase. Specimens were collected after termination in each phase.

### 4.2. Bronchioalveolar Lavage Fluid Analysis

After termination, bronchoalveolar lavage (BAL) fluid was collected as previously described [23]. Briefly, mice were sacrificed, and 3 × 800 μL of PBS was inserted into the trachea and re-aspirated via a syringe connected to a blunted 18G needle. The combined BAL fluid was thoroughly mixed, and 10 μL of the fluid was taken and mixed with 10 μL of Turk’s solution and inserted into a hemocytometer for manual counting of the cells. The remaining BAL fluid was then centrifuged at 15,000 rpm for 10 min. A total of 1500 μL was transferred to a different tube for storage, while the remaining amount was re-mixed. A total of 50 μL of the re-homogenized samples was then processed using Cytospin to produce a BAL fluid smear as per the manufacturer’s protocol. Staining of the BAL cells was performed using Diff-Quik (Sysmex, Kobe, Japan) as per the manufacturer’s protocol. Pictures of the cytology slides were taken using a Keyence BZ-X800 microscope, and the first 200 cells were counted, classified as either neutrophils, macrophages, or lymphocytes, and analyzed for differential cell count. 

### 4.3. Histological Analysis and Immunostaining

After the mice were sacrificed and perfused with cold PBS, the mouse lungs were fixed with 10% formalin for at least 24 h. After this period, lung specimens were processed into paraffin blocks and subsequently histological sections for further staining. The sections were first subjected to hematoxylin–eosin staining as previously described [22]. To analyze the mean linear intercept (MLI), at least three randomly selected images for each lung section were taken from 20×-magnification fields of view for each sample, averaged, and analyzed in ImageJ using the plugin “Measure MLI” [24]. We measured, in total, up to 18 images per experimental group (3 images per lung section × 3–6 lung sections per group).

For immunostaining, we performed immunohistochemistry staining using anti-CD-31 (1:100, CST) to measure the MVD and anti-JCAD (1:200, Invitrogen, Waltham, MA, USA) to localize JCAD in the lung sections as previously described. Briefly, after overnight antigen retrieval, blocking with 5% donkey serum in PBS-T, and overnight first antibody incubation and second antibody incubation were performed using the ImmPRESS HRP Polymer Detection Kit (VectorLabs, Newark, CA, USA) before DAB substrate (VectorLabs, Newark, CA, USA) application and mounting. At least three randomly selected 20×-magnification images were counted for the number of CD31-positive vessels from each lung section, averaged and treated as one sample each, and then compared between experimental groups to analyze the MVD [25]. We measured, in total, up to 18 images per experimental group (3 images per lung section × 3–6 lung sections per group). 

### 4.4. In Vitro Study

A549 was acquired from ATCC, while HPMECs were purchased from Takara. HPMECs were cultured in EGM-MV2 (Lonza, Basel, Switzerland) supplemented with the accompanying Bullet Kit until reaching ~90% confluency before silencing with KIAA1462 siRNA (Invitrogen, Waltham, MA, USA) or the negative control (Invitrogen, Waltham, MA, USA) using Lipofectamine RNAiMAX (Invitrogen, Waltham, MA, USA) as per the manufacturer’s protocol. After 24 h of siRNA treatment and a further 24 h of normal medium, the cells were then treated with TNFα (10 ng/mL) for 6 h before collection. For A549 cells, cells were cultured in RPMI-1640 (Gibco, Waltham, MA, USA) supplemented with 10% FBS (Gibco, Waltham, MA, USA) and 1% penicillin/streptomycin (Gibco, Waltham, MA, USA) until ~90% confluency before siRNA and TNFα treatments, similar to the HPMECs.

### 4.5. Quantitative Real-Time PCR Analysis 

Collected cell samples were harvested in Sepasol RNA I Super G (Nacalai Tesque, Kyoto, Japan), mixed with 20% chloroform to extract the RNA, and further processed into cDNA using the Primescript One-Step RT-PCR kit (Takara, Kusatsu, Shiga) as previously described [22]. Real-time qPCR was performed as previously described using the TAKARA ThermalCycler Dice Real Time System II at 95 °C for 15 s of denaturation, 60 °C for 20 s of annealing, and 72 °C for 30 s of extension for a total of 60 cycles before the final dissociation step (95 °C for 15 s followed by 60 °C for 30 s and a final 95 °C for 15 s of incubation). Cq data were quantified using ΔΔCq analysis with GAPDH as the reference gene. Primers are listed in Table 1.

### 4.6. Statistical Analysis

All quantitative data in this manuscript are presented as the mean ± standard error of mean (SEM). Statistical differences between two groups were analyzed using a two-tailed Student’s t-test. Statistical differences between three or more groups were analyzed using a one-way ANOVA test with Tukey’s post hoc test. A *p*-value of <0.05 was considered significant. All statistical analyses were performed using GraphPad Prism 10.3.1 (Activation number ca332c34-cef6-4dea-9013-5fdd3d4d48fa, GraphPad Software Inc., Boston, MA, USA).

## 5. Conclusions

The loss of KIAA1462/JCAD leads to the prevention of overt inflammatory and angiogenic responses of the lung, thereby ameliorating COPD development. Targeting KIAA1462/JCAD might hold potential for future COPD treatment. Further studies are needed to confirm its molecular mechanisms and its clinical feasibility.

## Figures and Tables

**Table 1:** List of primers used for qPCR analysis.

Primer Name	Sequence
GAPDH-Forward	5′-GCACCGTCAAGGCTGAGAAC-3′
GAPDH-Reverse	5′-ATGGTGGTGAAGACGCCAGT-3′
IL-6 Forward	5′-GGTACATCCTCGACGGCATCT -3′
IL-6 Reverse	5′-GTGCCTCTTTGCTGCTTTCAC-3′
RANTES-Forward	5′-AGCTTCCTTGAACCATTATGCTG-3′
RANTES-Reverse	5′-AGGTCTTCATTGGTGACCTGCT-3′
TNFα-Forward	5′-GCTTGTTCCTCAGCCTCTTC-3′
TNFα-Reverse	5′-GGTTATCTCTCAGCTCCACGC-3′
CXCL8-Forward	5′-GCATAAAGACATACTCCAAACC-3′
CXCL8-Reverse	5′ACTTCTCCACAACCCTCTG-3′
KIAA1462-Forward	5′-CCTGGAACTGGGAATGAGTATG-3′
KIAA1462-Reverse	5′-GTACTGAACGAAGCCGTCATAG-3′

## Data Availability

All data presented in this manuscript are available upon reasonable request.
